# Are There Still Difficult-to-Treat Patients with Chronic Hepatitis C in the Era of Direct-Acting Antivirals?

**DOI:** 10.3390/v14010096

**Published:** 2022-01-06

**Authors:** Paweł Pabjan, Michał Brzdęk, Magdalena Chrapek, Kacper Dziedzic, Krystyna Dobrowolska, Katarzyna Paluch, Anna Garbat, Piotr Błoniarczyk, Katarzyna Reczko, Piotr Stępień, Dorota Zarębska-Michaluk

**Affiliations:** 1 Department of Infectious Diseases, Voivodship Hospital, Jan Kochanowski University, 25-317 Kielce, Poland; pabjan3@tlen.pl (P.P.); katarzyna.pal@tlen.pl (K.P.); a.garbat@interia.pl (A.G.); diskstate@gmail.com (P.B.); reczko.katarzyna@poczta.fm (K.R.); p_stepien@interia.pl (P.S.); 2 Collegium Medicum, Jan Kochanowski University, 25-369 Kielce, Poland; michal.brzdek@gmail.com (M.B.); dzikac5@gmail.com (K.D.); krystyna.dobrowolska98@gmail.com (K.D.); 3 Faculty of Natural Sciences, Jan Kochanowski University, 25-369 Kielce, Poland; magdalena.chrapek@ujk.edu.pl

**Keywords:** hepatitis C, direct-acting antiviral, difficult-to-treat

## Abstract

Difficult-to-treat populations with chronic hepatitis C (CHC), in the era of interferon treatment, included patients with liver cirrhosis, kidney impairment, treatment-experienced individuals, and those coinfected with the human immunodeficiency virus. The current study aimed to determine whether, in the era of direct-acting antivirals (DAA), there are still patients that are difficult-to-treat. The study included all consecutive patients chronically infected with hepatitis C virus (HCV) who started interferon-free therapy between July 2015 and December 2020 in the Department of Infectious Diseases in Kielce. The analyzed real-world population consisted of 963 patients, and most of them were infected with genotype 1 (87.6%) with the predominance of subtype 1b and were treatment-naïve (78.8%). Liver cirrhosis was determined in 207 individuals (21.5%), of whom 82.6% were compensated. The overall sustained virologic response, after exclusion of non-virologic failures, was achieved in 98.4%. The univariable analysis demonstrated the significantly lower response rates in males, patients with liver cirrhosis, decompensation of hepatic function at baseline, documented esophageal varices, concomitant diabetes, body mass index ≥25, and previous ineffective antiviral treatment. Despite an overall very high effectiveness, some unfavorable factors, including male gender, genotype 3 infection, liver cirrhosis, and treatment experience, significantly reduce the chances for a virologic response were identified.

## 1. Introduction

Hepatitis C virus (HCV) infection, affecting, by the most recent estimations of the World Health Organization (WHO), approximately 71 million people globally, is one of the major public health issues [1,2]. Effective antiviral treatment is a first step to prevent the most serious complications of chronic hepatitis C (CHC), such as liver cirrhosis and hepatocellular carcinoma, resulting in nearly 400,000 deaths annually [1,3].

Six major viral genotypes have been identified that differ by more than 30% in genome structure, with GT1 being the most common in the world, followed by GT3 and 4 [4].

The standard therapy with pegylated interferon (pegIFN) and ribavirin (RBV) available from the beginning of the 20th century was successful in 50–80% of cases, depending on the genotype, the lowest efficacy being reported for GT1 and GT4, and the highest being for GT2 and GT3 [5,6,7,8].

Apart from infection with unfavorable genotypes, other patient-related factors were identified as lowering the effectiveness of antiviral therapy. Therefore, in the era of IFN treatment, we had the “difficult-to-treat” populations that responded poorly to therapy. They included patients with liver cirrhosis, kidney impairment, those with previous treatment failure, and individuals coinfected with human immunodeficiency virus (HIV). In some of these patients, such as those with cirrhosis or renal failure, safety issues related to contraindications to pegIFN + RBV therapy and treatment side effects, for which therapy was discontinued, also played a role [9]. In turn, patients with HIV coinfection were labeled difficult to treat due, not only, to low efficacy but also to a higher percentage of treatment ineligibility, nonadherence during the long treatment course, substance abuse, and medication intolerance [10].

The addition of the first direct-acting antivirals (DAA), from the class of protease inhibitors, telaprevir (TVR), and boceprevir (BOC) to the pegIFN + RBV regimen improved the efficacy in these groups of patients, as compared to the prior standard of care option, but the application of these drugs in real-world experience (RWE) settings resulted in lower effectiveness than in clinical trials, especially in a cirrhotic subpopulation, and was associated with increased rates of toxicity, which limited its use [11,12].

Triple therapy containing next-generation DAA, simeprevir (SMV), daclatasvir (DCV), or sofosbuvir (SOF), significantly improved the treatment response while reducing the therapy duration, but safety issues related to IFN remained [13,14,15]. The next step in the revolution of the therapy of chronic hepatitis C was the introduction of all-oral interferon-free regimens with the registration of pangenotypic options as the latest development. With this movement, the effectiveness of antiviral treatment increased substantially, exceeding 95%, and its duration was shortened to 8–12 weeks [16]. Importantly, the safety profile of therapy significantly improved, which meant that patients with CHC disqualified previously for IFN-based regimens due to contraindications, including cirrhotics, also decompensated, and renal impaired individuals, could receive treatment and be cured. This achievement has raised a hope to complete a goal, established by the WHO, to eliminate HCV as a major public threat by 2030 [1].

Our analysis aimed to assess the effectiveness and safety of chronic hepatitis C treatment with IFN-free regimens in RWE settings to determine whether, in the era of DAA therapy, we still have patients who are difficult to treat.

## 2. Materials and Methods

This observational study included all consecutive patients with chronic hepatitis C who started IFN-free DAA-based therapy between July 2015 and December 2020 in the Department of Infectious Diseases in Kielce. Data were collected retrospectively and submitted, following the National General Data Protection Regulation in Poland.

The drug use, dosage, and length of the treatment regimen were determined by treating physicians based on the applicable product characteristics and recommendations of the Polish Group of Experts for HCV, taking into account the criteria of the reimbursed therapeutic program of the National Health Fund [17,18,19].

The data collected at baseline were as follows: gender, age, body mass index (BMI), HCV genotype and viral load, the severity of liver disease, including fibrosis status, HCC, and liver transplantation history; additionally, in cirrhotic patients, the presence of esophageal varices, hepatic decompensation in the history and at the start of therapy were evaluated. Then, information regarding the presence of extrahepatic manifestations of HCV infection, as well as coinfections with human immunodeficiency virus (HIV) and hepatitis B virus (HBV), comorbidities and concomitant medications, and data on the previous antiviral treatment, similar to the current DAA regimen and laboratory parameters, including alanine transaminase (ALT) activity, bilirubin, creatinine and albumin concentration, hematology and coagulation findings, were captured. Hepatic fibrosis (F) was assessed non-invasively by real-time shear wave elastography (SWE) using the Aixplorer device (SuperSonic Imagine, Aix-en-Provence, France). Based on liver stiffness measurement, patients were assigned to the fibrosis stage from F0 to F4, according to the METAVIR score using the guidelines of the European Association for the Study of the Liver (EASL). The cut-off of 9 and 13 kilopascals was used for the prediction of F3 and F4, respectively [20].

Based on the clinical and laboratory data, patients with liver cirrhosis were scored on the Child–Pugh (CP) scale and Model of End-Stage Liver Disease (MELD). 

HCV RNA was assessed using the Xpert HCV Viral Load real-time assay with the lower limit of detection 10 IU/mL at the end of treatment (EOT), then, 12 weeks after therapy completion, and the negative result at this latter measurement was the efficacy endpoint defined as the sustained virologic response (SVR). Patients with no HCV RNA assessment, due to loss to follow-up (LTFU), were considered to be non-virologic failures, whereas those with detectable viremia were virologic non-responders. 

During the treatment and 12-week follow-up period the safety data were collected in terms of any modification, adverse events (AE), and deaths; in patients with liver cirrhosis, information on the AE of special interest, associated with deterioration of liver function (ascites, encephalopathy, and gastrointestinal bleeding), was submitted.

### Statistical Analysis

Continuous data were described by means, standard deviations, medians, and quartiles. Categorical data were summarized by frequencies and percentages. Group comparisons were performed using the chi-square or Fisher’s exact test for categorical variables and the Mann–Whitney test for continuous, non-normally distributed variables (normality of distribution was checked with the Shapiro–Wilk test).

Receiver operating characteristics (ROC) analysis was performed to assess whether continuous variables (age, BMI, ALT, bilirubin, albumin, creatinine, hemoglobin, platelets, HCV RNA) can be used to distinguish between responders and non-responders. For these variables, which were statistically able to distinguish between responders and non-responders, the optimal cut-off values were determined by maximizing Youden’s index.

Non-response to antiviral therapy was modeled by univariable logistic regression, and the odds ratios (OR) and 95% confidence intervals (95% CI) were calculated. A two-tailed *p*-value < 0.05 was considered statistically significant. All statistical analyses were performed using the R software package version 4.0.3.

## 3. Results

The analyzed RWE population consisted of 963 CHC patients, with a mean age of 50.4 ± 15.9 years, and was outnumbered by females (54.5%). Seventy-eight percent of them had coexisting diseases, with the most common being arterial hypertension, and 64.5% were treated with concomitant medications. Thirty-seven patients (3.8%) were diagnosed with kidney failure, nine of them had severe renal impairment, and six were dialyzed. The majority of patients were infected with GT1 (87.6%) with predominance of subtype 1b, followed by GT3 and 4 (Table 1). 

Most of the participants were treatment-naïve (78.8%), and among those, with a history of previous therapy, the relapse and non-response contributed in an equal proportion of 8%. Nearly half of the patients were diagnosed with minimal liver fibrosis while cirrhosis was determined in 207 individuals, corresponding to 21.5% of the cohort. Among them, 82.6% were assessed as compensated, and 15% and 2.4% were scored as B and C on the CP scale, respectively. The detailed characteristics of this population are presented in Table 2.

The majority of patients (65%) were assigned to genotype-specific regimens, with the most common option of ombitasvir/paritaprevir/ritonavir ± dasabivur ± ribavirin (OBV/PTV/r ± DSV ± RBV) (Table 3).

Among the pangenotypic regimens, the combination of glecaprevir/pibrentasvir (GLE/PIB) was used more frequently, as compared to sofosbuvir/velpatasvir (SOF/VEL) ± RBV, 22.1% and 12.9%, respectively. A total of 937 patients responded to the therapy, giving an overall SVR rate of 97.3% in the intent-to-treat analysis, and after the exclusion of 11 patients lost to follow-up (1.1%), 98.4% in the per-protocol analysis. Eight of 11 LTFU patients were not assessed for the treatment effectiveness due to death before 12 weeks after therapy completion. The efficacy rates achieved in the different therapeutic options, ranging from 93.9% to 100% in PP analysis, are presented in Table 4.

**Figure 1:**
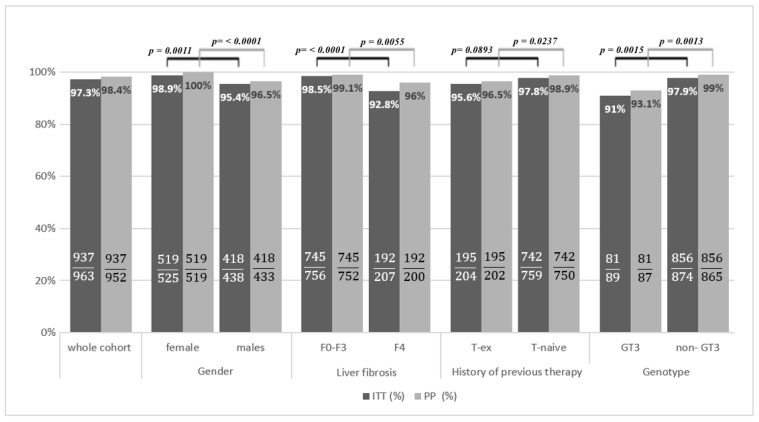
The comparison of SVR in the subpopulations. F: fibrosis; GT, genotype; ITT: intention-to-treat; PP: Per-protocol; SVR: sustained virologic response; T-ex: treatment experience; T-naive: treatment naive.

Patients with liver cirrhosis responded in a significantly lower percentage as compared to those with fibrosis F0–F3, both in ITT (92.8% vs. 98.5%, *p* < 0.0001) and PP analysis (96% vs. 99.1%, *p* = 0.0055). A worse response to the therapy was also obtained in treatment-experienced patients compared to treatment-naïve individuals, 95.6 vs. 97.8%, *p* = 0.0893, in the ITT and 96.5% vs. 98.9%, *p* = 0.0237, in PP analysis, respectively. The efficacy comparison carried out, taking into account the HCV genotype, revealed that patients infected with GT3 achieved a lower SVR than those infected with other genotypes, 91% vs. 97.9%, *p* = 0.0015, in the ITT and 93.1% vs. 99%, *p* = 0.0013, in PP analysis (Figure 1).

The gender of the patients also influenced the effectiveness; the SVR rate was significantly higher among females than males in ITT (95.4% vs. 98.9%, *p* = 0.0011) and PP analysis (96.5% vs. 100%, *p* < 0.0001). Thirty-five of 37 patients with kidney failure were treated successfully, while 2 were lost to follow-up, giving a response rate of 94.6% in ITT and 100% in PP analysis. Both HIV-coinfected patients achieved an SVR.

All 15 virologic non-responders were male (Table 5).

Significantly higher BMI (*p* = 0.0011), rates of GT3 infection (*p* = 0.0013) and liver cirrhosis (*p* = 0.0055), especially decompensated (*p* = 0.013), and percentage of patients with documented esophageal varices (*p* = 0.0104) were observed among non-responders as compared to those successfully treated. Among the baseline laboratory parameters, higher ALT activity (*p* = 0.002), higher bilirubin concentration (*p* = 0.0123), and lower platelet count (*p* = 0.0068) were noted in non-responders. Among patients who did not achieve viral clearance, a significantly higher proportion of treatment-experienced subjects (*p* = 0.0237), especially those after DAA-based therapy (*p* = 0.0008), was reported. 

In the univariable analysis BMI ≥ 25 kg/m^2^, infection with GT3, fibrosis F4 corresponding to liver cirrhosis, decompensation of liver function at baseline (B or C in CP scale), documented esophageal varices, concomitant diabetes, previous ineffective treatment, baseline ALT activity > 70 U/L, higher bilirubin concentration, and lower albumin level and platelet count were negative predictors of an SVR (Table 6).

The precise description of the 15 virologic non-responders with possible reasons for treatment failure is presented in Table 7.

Four of them were treatment-experienced males with liver cirrhosis infected with GT3. There were six patients with such characteristics in the entire database, and two remaining individuals responded to the therapy, so the likelihood of non-response in this specific subpopulation was 66.7% (4/6) compared to 1.1% (11/957) for those who did not have all of these four factors (*p* < 0.0001).

The majority of patients completed the treatment course as scheduled (97.3%). In 13 patients, RBV dosage was modified due to anemia, two inadherent patients experienced temporary treatment interruptions, and for 11 patients, therapy was permanently discontinued due to death (*n* = 3), adverse events (*n* = 3), by patient’s decision (*n* = 4), or for an unknown reason (*n* = 1). At least one AE was reported in 15.8% of patients, and the most frequent was weakness/fatigue, followed by anemia (Table 8). 

The safety profile was significantly worse in the cirrhotic subpopulation, with eight cases of deterioration of ascites, six emerging hepatic encephalopathy, and one gastrointestinal bleeding. All eight deaths and 17 of 26 serious adverse events were reported in patients with liver cirrhosis.

## 4. Discussion

In the current analysis, we confirmed the very high cure rate of CHC patients treated with all-oral DAA treatment across all therapeutic regimens, supporting conclusions from other RWE studies [21]. However, despite an overall SVR exceeding 98%, there are still patients who can be named difficult to treat and who are less likely to be cured. It should be noted that the scale of the phenomenon is much smaller than in the case of IFN-based therapies, and some patients historically considered difficult to treat can be treated effectively and safely in the era of DAA [22]. Such a population is patients with renal failure, including those on dialysis. In our study, they achieved an SVR of 100% in PP analysis. The kidney-impaired patients were treated with DAA regimens depending on the renal function; in those with severe kidney impairment, the ombitasvir/paritaprevir/ritonavir ± dasabuvir (OBV/PTV/r ± DSV) ± RBV, grazoprevir/elbasvir (GZR/EBR), or glecaprevir/pibrentasvir (GLE/PIB) combination without RBV was applied according to labels. Our data on the very high effectiveness of the DAA therapy, in patients with renal failure, are consistent with the results of clinical trials and RWE studies [2,3,4,5,6,7,8,9,10,11,12,13,14,15,16,17,18,19,20,21,22,23,24,25,26]. Importantly, in November 2019, the use of SOF-based options was approved in patients with advanced kidney disease, which enabled the management of those with concurrent decompensated liver cirrhosis, in whom a regimen containing a protease inhibitor is contraindicated, but there were no such individuals in our study [27].

Another group of patients who are no longer treatment refractory in the DAA era are those with HCV/HIV coinfection [28,29]. The population of such patients was too small in this analysis, only two individuals, but both responded to antiviral therapy.

While short treatment duration helps to maintain adherence and response rates are very high irrespective of the treatment regimen, it should be emphasized that potential interactions with antiretroviral agents should be investigated before treatment initiation to optimize DAA therapeutic options [20].

Patients with liver cirrhosis, who benefit from the introduction of DAA therapy due to a good safety profile, according to the results of the current study, are still harder to treat than others. The SVR of 96% in PP analysis, achieved in the analyzed group, predominantly composed of GT1b infected patients, is very high if we compare the response to IFN-based therapy, but it is significantly worse compared to patients without cirrhosis. Clinical trials evaluating the DAA in cirrhotic patients report efficacy at different levels, ranging from 85% to 100%, depending on the regimen used, the status of the cirrhosis (compensated or not), the history of previous therapy, type of GT, treatment duration, and possible RBV addition. The effectiveness achieved in patients with liver cirrhosis, infected with GT1 or GT4, following genotype-specific options ranged from 92% to 100% [30,31,32,33]. The clinical studies with pangenotypic regimens provided insight into treatment response in patients infected with all HCV genotypes, including GT3, which was second in frequency among those with liver cirrhosis in the current analysis.

According to published reports, the patients with compensated cirrhosis, treated with the GLE/PIB combination, achieved an overall SVR of 96% compared to a 98% response rate in the non-cirrhotic population [34]. However, even the ultimate cure rate of 100%, following a short 8-week regimen in treatment-naïve cirrhotics, was documented in the EXPEDITION-8 trial [35]. The cure rates of the SOF/VEL option, used in patients with compensated liver cirrhosis participating in ASTRAL-1 and -3 clinical trials, were 99% in those infected with GT1,2,4-6 and 91% in the case of GT3 infection, as compared to SVR of 99% and 97% in non-cirrhotic patients, respectively [36,37].

Many RWE studies confirm the very high cure rate following DAA therapy in patients with liver cirrhosis, but some of them also pointed out the difference in the effectiveness compared to non-cirrhotic patients [38,39,40]. Decompensation of liver function at baseline, defined as B or C in CP scale, was identified as an independent negative predictor of the SVR in our analysis, supporting results from clinical trials and RWE cohorts [41,42]. The presence of the esophageal varices, serving as a surrogate marker of clinically significant portal hypertension, has also been shown to be an independent negative prognostic factor of SVR in the current study. Due to irregular intrahepatic, splanchnic, and intestinal blood flow in patients with portal hypertension, the disturbances in pharmacokinetics, including modified drug uptake and distribution in hepatocytes, may reduce the treatment response [43].

Another unfavorable factor that reduced the chance of successful treatment, confirmed by the current study, was the history of previous therapy. The unsatisfactory effectiveness of 77% was achieved, especially in the group of DAA-experienced patients, and our findings on the worst therapeutic response in this subpopulation are consistent with other reports [44,45,46].

One of the possible factors responsible for the virologic failure, in patients treated previously with DAA, is a viral resistance [47]. Despite numerous real-world reports on DAA therapy, there are only a few papers that analyze therapeutic failure in the context of the presence of RAS, documenting its negative impact on the effectiveness [48,49,50,51]. The published data indicate that, in most patients who failed the IFN-free therapy, RAS are detectable within the target regions of the respective DAA classes, including inhibitors of the HCV protease (NS3), polymerase (NS5B), and replicase (NS5A) [47]. RAS, selected during therapy with NS3 and NS5B, disappear within a few weeks to months, except Q80K substitution in GT1a patients treated with NS3 inhibitors, while viral variants, emerging after NS5A-containing regimens, persist during long-term follow-up, even up to 4 years, depending on the type of drug and HCV genotype, thus exerting a greater influence on the effectiveness of the retreatment [47]. According to the most recent EASL guidelines, re-therapy of patients who did not respond to DAA regimen can be optimized based on resistance testing, if available, especially in those previously exposed on the NS5A inhibitors, in which the greatest effect on reducing the effectiveness was proven for the variant Y93H [47].

The available data indicate that the retreatment option that offers a chance for more effective therapy in prior DAA failure, including patients with Y93H substitution, is SOF/VEL/and voxilaprevir (VOX) combination, but this regimen was not available in our country within the therapeutic program in the analyzed period [47,52,53,54]. In the current study, 2 of 15 virologic nonresponders were treated previously with NS5A inhibitors, and both were diagnosed with compensated liver cirrhosis. One of them infected with GT1b retreated in the genotype-specific era and received LDV/SOF + RBV, and another infected with GT3 retreated in the era of availability of the pangenotypic drugs, received VEL/SOF + RBV, according to label.

Another group of patients identified as difficult-to-treat in this analysis were those infected with GT3, recognized previously easy-to-treat due to higher than in other genotype infections cure rate with IFN-based therapies. Among them, we observed an SVR of 93% compared to 99% in the non-GT3 population. At the beginning of the DAA era, the only IFN-free regimen available in Poland for this population was the SOF + RBV combination considered to be a suboptimal choice, while a more efficient option consisted of SOF and daclatasvir was not reimbursed and used in individual cases [55,56]. Although the SOF + RBV regimen was associated with an unsatisfactory virologic response, it had a good safety profile and, therefore, was used in the current study in GT3 infected patients with liver cirrhosis and contraindications to IFN who cannot wait for better options. At the same time, non-cirrhotic individuals were still treated with IFN-based therapies achieving a higher response rate. Despite the increased effectiveness of therapy after the introduction of highly potent pangenotypic drugs, the cure rate in GT3 infected patients is still inferior compared to other genotypes, and the difference is more pronounced in the presence of other factors, such as liver cirrhosis and history of previous therapy, and it was also confirmed by our study [46,57,58].

Among comorbidities, we identified the diabetes as an independent negative predictor of SVR. The two-way association between chronic hepatitis C and impaired glucose metabolism is well established; HCV infection triggers insulin resistance and diabetes, mostly type 2, and diabetes worsens the outcomes of hepatitis C, including higher risk of cirrhosis and primary liver cancer [59]. Although almost all attention is focused on the improvement of glucose metabolism after effective antiviral therapy, there are some studies from the interferon era confirming lower cure rate among patients with altered glucose metabolism [60].

The present analysis has several limitations related to its non-randomized design. Retrospective observational nature resulted in possible insufficient documentation of minor adverse events, electronic data capture with potential physician bias, and possible data entry errors. Some subpopulations were small, making it difficult to draw general conclusions.

According to criteria of the reimbursed therapeutic program of the National Health Fund, the resistance testing was not required before re-therapy. Thus, it was not assessed in the analysis.

However, the major strength of our study is the large number of included patients and the very high rate of those retained in the post-treatment evaluation, where only 11 individuals (1.1%) were lost to follow-up.

## 5. Conclusions

The current analysis confirmed the very high effectiveness and good safety profile of the antiviral therapy in RWE settings, across all DAA regimens, and revealed some unfavorable factors, such as male gender, infection with GT3, liver cirrhosis, and history of previous treatment, significantly reducing the chances for a virologic response.

## Figures and Tables

**Table 1:** Baseline Characteristics of HCV-Infected Patients Treated with IFN-Free Regimens.

Parameter	All Patients, *n* = 963
Gender, females/males, *n* (%)	525 (54.5)/438 (45.5)
Age [years] mean ± SD; min.–max.	50.4 ± 15.9; 19–89
Median (Q1, Q3)	50.0 (36.0, 63.0)
Females, age [years] mean ± SD; min.–max.	52.1 ± 16.3; 19–88
Median (Q1, Q3)	55.0 (36.0, 65.0)
Males, age [years] mean ± SD; min.–max.	48.4 ± 15.1; 19–89
Median (Q1, Q3)	45.0 (36.0, 60.0)
BMI [kg/m^2^] mean ± SD; min.–max.	25.9 ± 4.5; 15.6–45
Median (Q1, Q3)	25.4 (22.6, 28.5)
GT, *n* (%)	
1	34 (3.5)
1a	12 (1.3)
1b	797 (82.8)
2	0
3	89 (9.2)
4	29 (3)
5	0
6	2 (0.2)
Comorbidities, *n* (%)	
Any comorbidity	752 (78.1)
Hypertension	337 (35)
Diabetes	117 (12.1)
Renal disease	82 (8.5)
Kidney failure–eGRF < 30 mL/min, 30–60 mL/min	28 (2.9), 9 (0.9)
Dialysis	6 (0.6)
Autoimmune diseases	68 (7.1)
Non-HCC tumors	52 (5.4)
Other	663 (68.8)
Concomitant medications, *n* (%)	621 (64.5)
Liver fibrosis, *n* (%)	
F0	43 (4.5)
F1	472 (49)
F2	139 (14.4)
F3	102 (10.6)
F4	207 (21.5)
HCC history, *n* (%)	10 (1)
OLTx history, *n* (%)	4 (0.4)
HBV coinfection (HBsAg+), *n* (%)	7 (0.7)
HIV coinfection, *n* (%)	2 (0.2)
Extrahepatic manifestations of HCV, *n* (%)	
Cryoglobulinemia	446 (46.3)
Thyroid abnormalities with presence of anti-thyroid antibodies	86 (8.9)
Thrombocytopenia in noncirrhotics	38 (3.9)
Other	17 (1.8)
History of previous therapy, *n* (%)	
Treatment-naive	759 (78.8)
Non-responder	79 (8.2)
Relapser	78 (8.1)
Discontinuation due to safety reason	47 (4.9)
ALT IU/L, mean ± SD	72.4 ± 57.1
Median (Q1, Q3)	55.0 (35.0, 91.0)
Bilirubin mg/dL, mean ± SD	0.9 ± 1
Median (Q1, Q3)	0.8 (0.6, 1.0)
Albumin g/dL, mean ± SD	4 ± 0.4
Median (Q1, Q3)	4.1 (3.8, 4.3)
Creatinine mg/dL, mean ± SD	1 ± 0.6
Median (Q1, Q3)	0.9 (0.8, 1.0)
Hemoglobin g/dL, mean ± SD	14.3 ± 1.6
Median (Q1, Q3)	14.3 (13.3, 15.4)
Platelets, ×1000/µL, mean ± SD	184.8 ± 71.4
Median (Q1, Q3)	186.0 (140.0, 228.5)
HCV RNA × 10^6^ IU/mL, mean ± SD	2.8 ± 8.6
Median (Q1, Q3)	1.0 (0.3, 2.8)

ALT: alanine transaminase; BMI: body mass index; F: fibrosis; GT: genotype; HBsAg: hepatitis B surface antigen; HBV: hepatitis B virus; HCC: hepatocellular carcinoma; HCV: hepatitis C virus; HCV RNA: ribonucleic acid of hepatitis C virus; HIV: human immunodeficiency virus; IFN: interferon; OLTx: orthotopic liver transplantation; SD: standard deviation.

**Table 2:** Characteristics of 207 Cirrhotic Patients Infected with HCV Included in the Analysis.

Parameter	Patients with Liver Cirrhosis, *n* = 207
Gender, females/males, *n* (%)	97 (46.9)/110 (53.1)
Age [years] mean ± SD; min.–max.	59 ± 13.7; 21–89
Median (Q1, Q3)	60.0 (50.5, 69.0)
Females, age [years] mean ± SD; min.–max.	62.4 ± 12.7; 21–89
Median (Q1, Q3)	63.0 (56.0, 71.0)
Males, age [years] mean ± SD; min.–max.	56 ± 13.9; 21–89
Median (Q1, Q3)	57.0 (46.0, 65.8)
BMI [kg/m^2^] mean ± SD; min.–max.	27 ± 5.1; 17.5–44.9
Median (Q1, Q3)	26.6 (23.6, 29.7)
GT, *n* (%)	
1	2 (1)
1a	1 (0.5)
1b	169 (81.6)
2	0
3	32 (15.4)
4	2 (1)
5	0
6	1 (0.5)
Comorbidities, *n* (%)	
Any comorbidity	197 (95.2)
Hypertension	102 (49.3)
Diabetes	53 (25.6)
Renal disease	23 (11.1)
Autoimmune diseases	11 (5.3)
Non-HCC tumors	11 (5.3)
Other	185 (89.4)
Concomitant medications, *n* (%)	188 (90.8)
Diuretics, *n* (%)	92 (44.4)
History of hepatic decompensation, *n* (%)	
Ascites	28 (13.5)
Encephalopathy	11 (5.3)
Documented esophageal varices, *n* (%)	89 (43)
Hepatic decompensation at baseline, *n* (%)	
Moderate ascites–responded to diuretics	20 (9.7)
Tense ascites–not responded to diuretics	4 (1.9)
Encephalopathy	6 (2.9)
HCC history, *n* (%)	8 (3.9)
OLTx history, *n* (%)	0
Child-Pugh, *n* (%)	
A	171 (82.6)
B	31 (15)
C	5 (2.4)
MELD, *n* (%)	
<15	189 (91.3)
15–18	17 (8.2)
19–20	0
>20	1 (0.5)
HBV coinfection (HBsAg+), *n* (%)	3 (1.4)
HIV coinfection, *n* (%)	0
Extrahepatic manifestations of HCV, n (%)	
Cryoglobulinemia	132 (63.8)
Thyroid abnormalities with the presence of anti-thyroid antibodies	18 (8.7)
Other	2 (1)
History of previous therapy, *n* (%)	
Treatment-naive	133 (64.2)
Non-responder	37 (17.9)
Relapser	24 (11.6)
Discontinuation due to safety reason	13 (6.3)
Treatment regimens, *n* (%)	
ASV + DCV	5 (2.4)
LDV/SOF ± RBV	56 (27.1)
OBV/PTV/r ± DSV ± RBV	51 (24.6)
GZR/EBR	22 (10.6)
SOF + SMV ± RBV	4 (1.9)
SOF + RBV	18 (8.7)
SOF + DCV + RBV	1 (0.5)
GLE/PIB	21 (10.2)
SOF/VEL ± RBV	29 (14)
ALT IU/L, mean ± SD	97.2 ± 68.3
Median (Q1, Q3)	78.0 (48.5, 127.0)
Bilirubin mg/dL, mean ± SD	1.5 ± 1.8
Median (Q1, Q3)	1.2 (0.9, 1.7)
Albumin g/dL, mean ± SD	3.6 ± 0.5
Median (Q1, Q3)	3.6 (3.3, 3.9)
Creatinine mg/dL, mean ± SD	0.9 ± 0.3
Median (Q1, Q3)	0.9 (0.8, 1.0)
Hemoglobin g/dL, mean ± SD	13.6 ± 1.7
Median (Q1, Q3)	13.8 (12.4, 14.6)
Platelets, ×1000/µL, mean ± SD	108.7 ± 57.4
Median (Q1, Q3)	97.0 (71.0, 136.5)
HCV RNA × 10^6^ IU/mL, mean ± SD	1.9 ± 5.2
Median (Q1, Q3)	0.5 (0.1, 1.5)

ALT: alanine transaminase; ASV: asunaprevir; BMI: body mass index; DCV: daclatasvir; DSV: dasabuvir; EBR: elbasvir; GLE: glecaprevir; GT: genotype; GZR: grazoprevir; HBsAg: hepatitis B surface antigen; HBV: hepatitis B virus; HCC: hepatocellular carcinoma; HCV: hepatitis C virus; HCV RNA: ribonucleic acid of hepatitis C virus; HIV: human immunodeficiency virus; LDV: ledipasvir; MELD: Model End-Stage Liver Disease; OBV: ombitasvir; OLTx: orthotopic liver transplantation; PIB: pibrentasvir; PTV/r: paritaprevir; RBV: ribavirin; SD: standard deviation; SMV: simeprevir; SOF: sofosbuvir; VEL: velpatasvir.

**Table 3:** Therapeutic Regimens Used in the Analyzed Population.

Parameter	HCV-Infected Patients *n* = 963
Genotype-specific treatment regimens (*n* = 626), *n* (%)	
ASV + DCV	19 (2)
LDV/SOF ± RBV	178 (18.5)
OBV/PTV/r ± DSV ± RBV	233 (24.2)
GZR/EBR ± RBV	156 (16.2)
SOF + SMV ± RBV	4 (0.4)
SOF + RBV	34 (3.5)
SOF + DCV ± RBV	2 (0.2)
Pangenotypic regimens (*n* = 337), *n* (%)	
GLE/PIB	213 (22.1)
SOF/VEL ± RBV	124 (12.9)

ASV: asunaprevir; DCV: daclatasvir; DSV: dasabuvir; EBR: elbasvir; GLE: glecaprevir; GZR: grazoprevir; HCV: hepatitis C virus; LDV: ledipasvir, OBV: ombitasvir, PIB: pibrentasvir; PTV/r: paritaprevir; RBV: ribavirin; SMV: simeprevir; SOF: sofosbuvir; VEL: velpatasvir.

**Table 4:** Treatment Efficacy According to Therapeutic Regimen.

Regimen/Efficacy	ASV + DCV	GLE/PIB	LDV/SOF ± RBV	OBV/PTV/r ± DSV ± RBV	GZR/EBR	SOF + SMV ± RBV	SOF + RBV	SOF/VEL ± RBV	SOF + DCV ± RBV
SVR ITT, *n* (%)	16/19 (84.2)	209/213 (98.1)	173/178 (97.2)	229/233 (98.3)	152/156 (97.4)	4/4 (100)	31/34 (91.2)	121/124 (97.6)	2/2 (100)
SVR PP, *n* (%)	16/17 (94.1)	209/213 (98.1)	173/175 (98.9)	229/232 (98.7)	152/153 (99.3)	4/4 (100)	31/33 (93.9)	121/123 (98.4)	2/2 (100)

ASV: asunaprevir; DCV: daclatasvir; DSV: dasabuvir; EBR: elbasvir; GLE: glecaprevir; GZR: grazoprevir; ITT: intention-to-treat; LDV: ledipasvir; OBV: ombitasvir; PIB: pibrentasvir; PP: Per-protocol; PTV/r: paritaprevir; RBV: ribavirin; SMV: simeprevir; SOF: sofosbuvir; SVR: sustained virologic response; VEL: velpatasvir.

**Table 5:** The Comparison of Virological Responders and Non-Responders to Antiviral Therapy.

Parameter	Responders *n* = 937	Non-Responders *n* = 15	*p* Value
Gender, females/males, *n* (%)	519 (55.4)/418 (44.6)	0/15 (100)	<0.0001
Age [years] mean ± SD; min.–max.	50.3 ± 15.9; 19–89	49.3 ± 8.2; 31–59	
Median (Q1, Q3)	50 (36, 63)	52.0 (46.0, 55.5)	0.842
Females, age [years] mean ± SD; min.–max.	51.9 ± 16.4; 19–88	NA	
Median (Q1, Q3)	55 (36, 65)		NA
Males, age [years] mean ± SD; min.–max.	48.2 ± 15.2; 19–89	49.3 ± 8.2; 31–59	
Median (Q1, Q3)	45 (36, 60)	52.0 (46.0, 55.5)	0.5557
BMI [kg/m^2^] mean ± SD; min.–max	25.8 ± 4.5; 15.6–45	29.4 ± 3.6; 24.9–36.3	
Median (Q1, Q3)	25.3 (22.6, 28.4)	28.4 (27.1, 30.2)	0.0011
GT, *n* (%)			0.0105
1	34 (3.6)	0	
1a	12 (1.3)	0	
1b	780 (83.3)	8 (53.3)	
2	0	0	
3	81 (8.6)	6 (40)	
4	28 (3)	1 (6.7)	
5	0	0	
6	2 (0.2)	0	
GT 3, *n* (%)	81 (8.6)	6 (37.5)	0.0013
GT 4, *n* (%)	28 (3.0)	1 (6.7)	0.3735
Comorbidities, *n* (%)			
Any comorbidity	728 (77.7)	13 (86.5)	0.5424
Hypertension	328 (35)	3 (20)	0.226
Diabetes	107 (11.4)	6 (40)	0.005
Renal disease	78 (8.3)	2 (13.3)	0.3636
Autoimmune diseases	67 (7.2)	0	0.6171
Non-HCC tumors	51 (5.4)	0	1
Other	641 (68.4)	12 (80)	0.4131
Kidney failure, *n* (%)	35 (3.7)	0	1
Concomitant medications, *n* (%)	598 (63.8)	12 (80)	0.1951
Liver fibrosis, *n* (%)			0.0776
F0	43 (4.6)	0	
F1	465 (49.6)	6 (40)	
F2	136 (14.5)	1 (6.7)	
F3	101 (10.8)	0	
F4	192 (20.5)	8 (53.3)	
Liver fibrosis F4, *n* (%)	192 (20.5)	8 (53.3)	0.0055
History of previous therapy, *n* (%)			0.0237
Treatment-naive	742 (79.2)	8 (53.3)	
Treatment-experienced	195 (20.8)	7 (46.7)	
DAA-experienced patients, *n* (%)	10 (1.1)	3 (20)	0.0008
History of hepatic decompensation, *n* (%)			
Ascites	27 (2.9)	1 (6.7)	0.3631
Encephalopathy	10 (1.1)	1 (6.7)	0.1611
Documented esophageal varices, *n* (%)	87 (9.3)	5 (33.3)	0.0104
Hepatic decompensation at baseline, *n* (%)			
Moderate ascites–responded to diuretics	19 (2)	0	1
Tense ascites–not responded to diuretics	3 (0.3)	0	1
Encephalopathy	5 (0.5)	0	1
HCC history, *n* (%)	9 (1)	0	1
OLTx history, *n* (%)	4 (0.4)	0	1
Child-Pugh B or C, *n* (%)	30 (3.2)	3 (20)	0.013
HBV coinfection (HBsAg+), *n* (%)	7 (0.7)	0	1
HIV coinfection, *n* (%)	2 (0.2)	0	1
Extrahepatic manifestations of HCV, *n* (%)			
Cryoglobulinemia	431 (46)	9 (60)	0.2805
Thyroid abnormalities with presence of anti-thyroid antibodies	85 (9.1)	0	0.3862
Thrombocytopenia in noncirrhotics	38 (4.1)	0	1
ALT IU/L, mean ± SD	71.8 ± 57.2	96 ± 34.3	
Median (Q1, Q3)	53.0 (35.0, 90.0)	91.0 (70.0, 108.5)	0.002
Bilirubin mg/dL, mean ± SD	0.9 ± 1	1.2 ± 0.6	
Median (Q1, Q3)	0.8 (0.6, 1.0)	1.1 (0.8, 1.3)	0.0123
Albumin g/dL, mean ± SD	4 ± 0.4	3.8 ± 0.6	
Median (Q1, Q3)	4.1 (3.8, 4.3)	3.9 (3.5, 4.0)	0.0511
Creatinine mg/dL, mean ± SD	1 ± 0.6	1 ± 0.2	
Median (Q1, Q3)	0.9 (0.8, 1.0)	0.9 (0.8, 1.0)	0.5054
Hemoglobin g/dL, mean ± SD	14.3 ± 1.6	14.6 ± 1.4	
Median (Q1, Q3)	14.3 (13.3, 15.4)	14.6 (14.4, 15.5)	0.2688
Platelets, ×1000/µL, mean ± SD	186.2 ± 71.1	132.9 ± 60.6	
Median (Q1, Q3)	187.0 (142.0, 230.0)	114.0 (77.0, 193.0)	0.0068
HCV RNA × 10^6^ IU/mL, mean ± SD	2.9 ± 8.7	2.4 ± 2.7	
Median (Q1, Q3)	1.0 (0.3, 2.8)	1.5 (0.5, 3.8)	0.3051

ALT: alanine transaminase; BMI: body mass index; DAA: direct-acting antivirals; F: fibrosis; GT: genotype; HBsAg: hepatitis B surface antigen; HBV: hepatitis B virus; HCC: hepatocellular carcinoma; HCV: hepatitis C virus; HCV RNA: ribonucleic acid of hepatitis C virus; HIV: human immunodeficiency virus; NA: not applicable (there were no women among nonresponders); OLTx: orthotopic liver transplantation; SD: standard deviation.

**Table 6:** Univariable Predictors of Non-Response to Antiviral Therapy.

Parameter		Univariable OR	95% CI	*p* Value
Age		1	0.96–1.03	0.8187
BMI		1.15	1.05–1.25	0.0031
BMI	<25	Ref. level		
	25 or more	12.83	1.68–97.88	0.0139
Genotype 3	no	Ref. level		
	yes	7.05	2.45–20.29	0.0003
Genotype 4	no	Ref. level		
	yes	2.32	0.29–18.25	0.4243
Any comorbidity	no	Ref. level		
	yes	1.87	0.42–8.33	0.4139
Hypertension	no	Ref. level		
	yes	0.46	0.13–1.66	0.2371
Diabetes	no	Ref. level		
	yes	5.17	1.81–14.81	0.0022
Renal disease	no	Ref. level		
	yes	1.69	0.38–7.64	0.4928
Autoimmune diseases	no	Ref. level		
	yes	NA (0 in cell)		
Non-HCC tumors	no	Ref. level		
	yes	NA (0 in cell)		
Other comorbidity	no	Ref. level		
	yes	1.85	0.52–6.59	0.3446
Concomitant medications	no	Ref. level		
	yes	2.27	0.64–8.09	0.2072
Liver fibrosis	F0	Ref. level		
	F1	NA (0 in cell)		
	F2	NA (0 in cell)		
	F3	NA (0 in cell)		
	F4	NA (0 in cell)		
Liver fibrosis, F4	no	Ref. level		
	yes	4.43	1.59–12.38	0.0045
Ascites	no	Ref. level		
	yes	2.41	0.31–18.97	0.4043
Encephalopathy	no	Ref. level		
	yes	6.62	0.79–55.29	0.0809
Documented esophageal varices	no	Ref. level		
	yes	4.89	1.63–14.62	0.0046
Moderate ascites at baseline	no	Ref. level		
	yes	NA (0 in cell)		
Tense ascites at baseline	no	Ref. level		
	yes	NA (0 in cell)		
Encephalopathy at baseline	no	Ref. level		
	yes	NA (0 in cell)		
HCC history	no	Ref. level		
	yes	NA (0 in cell)		
OLTx history	no	Ref. level		
	yes	NA (0 in cell)		
MELD	14 or less	Ref. level		
	15–18	NA (0 in cell)		
	19–20	NA (0 in cell)		
	21 or more	NA (0 in cell)		
Child Pugh B or C	no	Ref. level		
	yes	7.56	2.03–28.19	0.0026
HBV coinfection HBsAg plus	no	Ref. level		
	yes	NA (0 in cell)		
HIV coinfection	no	Ref. level		
	yes	NA (0 in cell)		
Cryoglobulinemia	no	Ref. level		
	yes	1.76	0.62–4.99	0.2866
History of previous therapy	Treatment-naive	Ref. level		
	Nonresponder	2.44	0.51–11.7	0.2645
	Relapser	5.01	1.47–17.05	0.0098
	Discontinuation due to safety reason	2.06	0.25–16.84	0.4998
History of previous therapy	Treatment naive	Ref. level		
	Treatment experienced	3.33	1.19–9.29	0.0216
Therapy	AS + DCV	Ref. level		
	LDV/SOF +/− RBV	0.18	0.02–2.15	0.1778
	OBV/PTV/r +/− DSV +/− RBV	0.21	0.02–2.13	0.1867
	GZR/EBR +/− RBV	0.11	0.01–1.76	0.1176
	SOF + SMV +/− RBV	NA (0 in cell)		
	SOF + DCV +/− RBV	NA (0 in cell)		
	SOF + RBV	1.03	0.09–12.27	0.9799
	GLE/PIB	0.31	0.03–2.9	0.3025
	SOF/VEL +/− RBV	0.26	0.02–3.08	0.2886
ALT		1	1–1.01	0.1092
ALT	<70	Ref. level		
	70 or more	4.85	1.53–15.35	0.0072
Bilirubin		1.12	0.88–1.43	0.3501
Bilirubin	<0.98	Ref. level		
	0.98 or more	5.07	1.72–14.98	0.0033
Albumin		0.31	0.12–0.85	0.0231
Creatinine		0.98	0.39–2.42	0.9596
Hemoglobin		1.14	0.82–1.59	0.4286
Platelets		0.99	0.98–1	0.0045
PLT	<115	Ref. level		
	115 or more	0.17	0.06–0.47	0.0007
HCV RNA		0.99	0.91–1.08	0.8547

ALT: alanine transaminase; ASV: asunaprevir; BMI: body mass index; DCV: daclatasvir; DSV: dasabuvir; EBR: elbasvir; F: fibrosis; GLE: glecaprevir; GZR: grazoprevir; HBsAg: hepatitis B surface antigen; HBV: hepatitis B virus; HCC: hepatocellular carcinoma; HCV RNA: ribonucleic acid of hepatitis C virus; HIV: human immunodeficiency virus; LDV: ledipasvir, NA: not available; OBV: ombitasvir, OLTx,: orthotopic liver transplantation; RBV: ribavirin; Ref.: reference; SMV: simeprevir; SOF: sofosbuvir; PIB: pibrentasvir; PTV/r: paritaprevir; VEL: velpatasvir.

**Table 7:** Characteristics of 15 Virologic Failures to Treatment.

Patient	Age	GT	F, CP	Regimen	History of Previous Therapy	Baseline HCV RNA × 10^6^ IU/mL	Treatment Course	EOT	Comment (Possible Reason of Failure)
Male 1	48	1B	1	GZR/EBR, 12 wks	treatment-naive	6.07	according to schedule	TND	
Male 2	44	1B	1	OBV/PTV/r + DSV, 8 wks	treatment-naive	1.53	according to schedule	TND	
Male 3	56	1B	2	ASV + DCV, 24 wks	treatment-naive	1.59	according to schedule	TD	
Male 4	59	1B	4, CP-A	OBV/PTV/r + DSV + RBV, 12 wks	non-responder (TVR + pegIFN + RBV)	0.58	according to schedule	TD	liver cirrhosis, non-response to previous therapy, liver cirrhosis
Male 5	59	1B	4, CP-A	LDV/SOF + RBV, 12 wks	relapser (OBV/PTV/r + DSV + RBV)	1.65	according to schedule	TND	liver cirrhosis, non-response to the previous therapy, liver cirrhosis
Male 6	54	1B	4, CP-B	LDV/SOF + RBV, 12 wks	treatment-naive	0.25	according to schedule	TND	decompensated liver cirrhosis
Male 7	31	1B	1	GLE/PIB, 8 wks	treatment-naive	0.53	modified	TD	no adherence (irregular use of the drug due to alcohol abuse)
Male 8	48	1B	4, CP-B	VEL/SOF + RBV, 12 wks	treatment-naive	0.31	according to schedule	TND	decompensated liver cirrhosis
Male 9	54	3	4, CP-A	GLE/PIB, 16 wks	relapser (SOF + RBV)	4.03	according to schedule	TND	liver cirrhosis, non-response to previous therapy
Male 10	48	4	1	OBV/PTV/r + RBV, 12 wks	relapser (SMV + PegIFN + RBV)	3.6	according to schedule	TND	liver cirrhosis, non-response to previous therapy, no adherence to the current treatment
Male 11	55	3	4, CP-B	SOF + RBV, 24 wks	non-responder (PegIFN + RBV)	0.43	according to schedule	TND	decompensated liver cirrhosis, non-response to previous therapy
Male 12	52	3	4, CP-A	SOF + RBV, 24 wks	discontinuation due to safety reason (IFN + RBV)	0.5	according to schedule	TND	liver cirrhosis
Male 13	56	3	4, CP-A	VEL/SOF + RBV, 24 wks	relapser (GLE/PIB)	1.08	according to schedule	TND	liver cirrhosis, non-response to the previous therapy
Male 14	34	3	1	GLE/PIB, 8 wks	treatment-naive	10.0	according to schedule	TND	
Male 15	42	3	1	GLE/PIB, 8 wks	treatment-naive	4.57	according to schedule	TND	

ASV: asunaprevir; CP: Child–Pugh scale; DCV: daclatasvir; DSV: dasabuvir; EBR: elbasvir; EOT: end of treatment; F: fibrosis; GLE: glecaprevir; GT: genotype; GZR: grazoprevir; HCV RNA: hepatitis C virus ribonucleic acid; LDV: ledipasvir; OBV: ombitasvir; pegIFN: pegylated interferon; PIB: pibrentasvir; PTV/r: paritaprevir; RBV: ribavirin; SMV: simeprevir; SOF: sofosbuvir; TD: target detected; TND: target not detected; TVR: telaprevir; VEL: velpatasvir; wks, weeks.

**Table 8:** Safety of DAA-Based Therapy.

Parameter	All Patients *n* = 963	Non-Cirrhotics *n* = 756	Cirrhotics *n* = 207	*p* Value
Treatment course, *n* (%)				
according to schedule	937 (97.3)	745 (98.6)	192 (92.8)	<0.0001
therapy modification	15 (1.6) **^1^**	7 (0.9) **^6^**	8 (3.9) **^9^**	
therapy discontinuation	11 (1.1) **^2^**	4 (0.5) **^7^**	7 (3.4) **^10^**	
Patients with at least one AE, *n* (%)	152 (15.8)	75 (9.9)	77 (37.2)	<0.0001
Serious adverse events, *n* (%)	26 (2.7) **^3^**	9 (1.2) **^8^**	17 (8.2) **^11^**	<0.0001
AEs leading to treatment discontinuation, *n* (%)	3 (0.3) **^4^**	0	3 (1.4) **^12^**	0.01
Most common AEs (≥2%), *n* (%)				
weakness/fatigue	54 (5.6)	30 (4)	24 (11.6)	<0.0001
anemia	33 (3.4)	8 (1.1)	25 (12.1)	<0.0001
AEs of particular interest, *n* (%)				
Ascites	8 (0.8)	NA	8 (3.9)	<0.0001
hepatic encephalopathy	6 0.6)	NA	6 (2.9)	<0.0001
gastrointestinal bleeding	1 (0.1)	NA	1 (0.5)	0.215
Death, *n* (%)	8 (0.8) **^5^**	0	8 (3.9)	<0.0001

As shown: **^1^** 13 × RBV dosage modification, 2 × nonadherence (temporary treatment interruptions); **^2^** 3 × AEs, 3 × death, 4 × patient’s decision, 1 unknown reason; **^3^** encephalopathy, fracture of lower extremity, dysplastic nodules in the liver, hepatocellular carcinoma, balance disorders, diarrhea, interstitial lung disease, 2 liver impairment, acute hepatitis, ALT elevation, gastrointestinal bleeding, severe thrombocytopenia, progression of hepatocellular carcinoma, ptosis of the left eyelid, liver transplantation, lung cancer, 4 cerebral stroke, clostridium difficile infection, arterial hypertension, head injury, COVID-19, 1 × (myocardial infarction, pulmonary embolism); **^4^** acute hepatitis, vomits, cerebral stroke; **^5^** 2 × liver impairment, 2 × hepatocellular carcinoma, cerebral stroke, COVID-19, cardiac arrest; **^6^** 5 × RBV dosing, 2 × no adherence; **^7^** 4 × non-adherence; **^8^** balance disorders, diarrhea, ALT elevation, ptosis of the left eyelid, lung cancer, 2 cerebral stroke, 1 × myocardial infarction with pulmonary embolism; **^9^** 8 × RBV dosing; **^10^** 3 × AEs, 3 × death, 1 unknown reason; **^11^** encephalopathy, fracture of lower extremity, dysplastic nodules in the liver, hepatocellular carcinoma, interstitial lung disease, 2 liver impairment, acute hepatitis, gastrointestinal bleeding, severe thrombocytopenia, progression of hepatocellular carcinoma, liver transplantation, 2 cerebral stroke, clostridium difficile infection, head injury, COVID-19; **^12^** acute hepatitis, vomits, and cerebral stroke. DAA: direct-acting antivirals; AE: adverse event; NA: not available.

## Data Availability

Data supporting reported results can be provided upon request from the corresponding author.
